# Propagation Model of Panic Buying Under the Sudden Epidemic

**DOI:** 10.3389/fpubh.2021.675687

**Published:** 2021-04-22

**Authors:** Peihua Fu, Bailu Jing, Tinggui Chen, Chonghuan Xu, Jianjun Yang, Guodong Cong

**Affiliations:** ^1^School of Management and E-Business, Zhejiang Gongshang University, Hangzhou, China; ^2^School of Statistics and Mathematics, Zhejiang Gongshang University, Hangzhou, China; ^3^School of Business Administration, Zhejiang Gongshang University, Hangzhou, China; ^4^Department of Computer Science and Information Systems, University of North Georgia, Atlanta, GA, United States; ^5^School of Tourism and Urban-Rural Planning, Zhejiang Gongshang University, Hangzhou, China

**Keywords:** panic buying, group decision-making, sudden epidemic, behavior spread, propagation model

## Abstract

The sudden outbreak of COVID-19 at the end of 2019 has had a huge impact on people's lives all over the world, and the overwhelmingly negative information about the epidemic has made people panic for the future. This kind of panic spreads and develops through online social networks, and further spreads to the offline environment, which triggers panic buying behavior and has a serious impact on social stability. In order to quantitatively study this behavior, a two-layer propagation model of panic buying behavior under the sudden epidemic is constructed. The model first analyzes the formation process of individual panic from a micro perspective, and then combines the Susceptible-Infected-Recovered (SIR) Model to simulate the spread of group behavior. Then, through simulation experiments, the main factors affecting the spread of panic buying behavior are discussed. The experimental results show that: (1) the dissipating speed of individual panics is related to the number of interactions and there is a threshold. When the number of individuals involved in interacting is equal to this threshold, the panic of the group dissipates the fastest, while the dissipation speed is slower when it is far from the threshold; (2) The reasonable external information release time will affect the occurrence of the second panic buying, meaning providing information about the availability of supplies when an escalation of epidemic is announced will help prevent a second panic buying. In addition, when the first panic buying is about to end, if the scale of the second panic buying is to be suppressed, it is better to release positive information after the end of the first panic buying, rather than ahead of the end; and (3) Higher conformity among people escalates panic, resulting in panic buying. Finally, two cases are used to verify the effectiveness and feasibility of the proposed model.

## Introduction

At the end of 2019, COVID-19 swept the world, causing many panic buying behaviors. At present, the long-term epidemic and the overwhelmingly negative news have made people panic about the future. Driven by this panic, panic buying has been rampant everywhere. For example, there has been news that toilet paper and masks are the same raw material, and the shortage of masks will inevitably lead to a shortage of toilet paper, which has triggered a panic buying of toilet paper in Japan, Australia, and other places (1). The panic buying boom quickly spread to more people through social media, and further amplified people's panic about the shortage of materials, and resulted in offline large-scale panic buying. Therefore, panic buying not only seriously endangers social order and environmental safety, but also easily causes insufficient social supply. Therefore, it is of important theoretical and practical significance to analyze the key factors affecting panic buying and explore the underlying reasons for its formation.

Arafat et al. (2) believes that panic buying may refer to the phenomenon of a recent increase in business of one or more essential goods in excess of regular need promoted by advertisement, usually after a disaster or an outbreak, resulting in an imbalance between supply and demand. Tahir et al. (3) also mentioned that panic buying usually occurs after consumers face or perceive disasters. After the occurrence of COVID-19, scholars have generally observed the impact of COVID-19 on commodity supply and need (4) and public mental health (5), which has led to a surge in panic buying incidents around the world (6). Herd mentality promotes the further spread of panic buying behavior (7). However, current research is mostly qualitative explanations without quantitative analysis. In addition, most of the research methods are statistical analysis methods, such as stepwise regression (8) or structural equation (9). This type of method only reflects historical conditions and cannot reflect changes in real conditions, and cannot restore the panic buying phenomenon or study it from appearance through to development to disappearance.

The suddenness and severity of sudden disaster events will trigger people's ultra-large-scale need for basic materials in the short term, which will cause a certain impact on the supply chain of survival necessities such as food, and even interrupt the supply chain. The ensuing imbalance of supply and need has further aggravated the panic buying behavior of people. Forbes (10) took the Christchurch earthquake in 2011 as an example, and deeply studied the change of consumer preferences caused by disasters; they found that consumers bought more practical products necessary for survival after disasters. Upton and Nuttall (11) proposed an agent-based model to simulate the transient need of the supply chain and consumers under the fuel crisis event and verified it with the fuel panic crisis events in the UK in 2000 and 2012, which provided practical suggestions for the panic buying of fuel. Arafat et al. collected media reports with the keyword of 'panic buying' (2), and found through statistical data analysis (12) that the sense of product scarcity was an important factor leading to panic buying during COVID-19. In addition, there were also factors such as increased demand, importance of products, anticipation of price hike, etc. To investigate the mechanism of urban consumers' food hoarding behaviors, Wang and Holly (13) took three cities in China as samples and used the multivariate probit model to study. They found that people's food on hand and their expectation of the possibility of COVID-19 infection were the main factors affecting food hoarding. The above literature explains the occurrence of panic buying from the perspective of imbalance between supply and need. After a sudden epidemic such as COVID-19, the public's need for food and other practical commodities surged. Due to insufficient market supply, commodity shortages and price increases have occurred, intensifying panic buying behavior. Compared with other disasters, the particularity of COVID-19, that is, the risk of contracting the virus, will have an important impact on people's panic buying behavior. Therefore, when studying panic buying behavior under the sudden epidemic, it is necessary to consider both physiological (material) and safety needs.

Sudden disaster events often trigger negative emotions among people. Some scholars have interpreted panic buying behavior from an emotional perspective. Thomas and Monica (14) took the September 11 attacks in the United States as an example, and pointed out that panic buying was a kind of self-protection behavior taken by the public in response to terrorism in panic. Sneath et al. (15) took Hurricane Katrina in 2005 as an example, and proposed a structural model based on the life event theory. The results showed that event-induced stress affects depression, which in turn leads to impulsive and compulsive buying behavior. Based on the stimulus-body response (SOR) model, Pandita et al. (16) adopted qualitative research methods, such as personal interview, and found that COVID-19 would lead to students' psychological problems, such as academic anxiety and fear, and behavioral problems such as panic buying. Bacon and Corr (17) conducted a questionnaire survey of British respondents and found that people were experiencing a psychological conflict between the urge to stay safe and the desire to maintain a normal, pleasurable life, and panic buying was one of the ways to improve this psychological conflict. Christian and Ronn (18) used the health anxiety scale and open-ended questions to conduct online surveys on people's feelings, thoughts, and behaviors during the period of strengthening community isolation, and constructed the spectrum of panic consequences caused by COVID-19, including panic buying (18). Yuen et al. (19) systematically reviewed the psychological causes of panic buying and pointed out that people would regard panic buying as the behavior of relieving anxiety and re-controlling a crisis. Jezewska et al. (20) used logistic regression analysis on the data of 1,033 Polish adults, and found that stress and trust in different information sources can lead to people's fear of limited food, and then lead to panic buying behavior. Arafat et al. (21) systematically reviewed the psychological explanations behind panic buying in critical moments and found that fear of scarcity and losing control over the environment, insecurity (which could be because of fear), social learning, and exacerbation of anxiety, are the basic primitive responses of humans responsible for the panic buying phenomenon. The above literature describes the emotional state of people's panic, anxiety, and depression after a disaster and the aggravating effect of these emotions on panic buying behavior. However, the research methods are mostly qualitative research, such as questionnaires and interviews, and there is limited research on the quantitative relationship between emotion and behavior. At the same time, the existing quantitative relationship research does not consider the influence of commodity supply and need on emotion.

Panic buying behavior is easy to spread in social groups, and this behavior spreading phenomenon is closely related to people's herd psychology. Charles (22), a British scholar, used a large number of factual cases to show that when an individual was in a group environment, he was quick to show extreme imitation and gregariousness. Pochea et al. (23) used quantile regression analysis as an estimation method, and found evidence of herding behavior in all central and Eastern European countries, except Poland and Romania: when the market rises, investors will follow each other in buying transactions, but when the market turns down, investors will not follow each other. Ahmed et al. (24) used the multivariate method based on the structural equation model to study the data of 889 consumers and found that peer purchase and other factors had an important impact on the impulsive purchase mode. Zheng et al. (25) pointed out that consumers tend to imitate others, and social media posts can play an important role in the diffusion of imitation and purchase behavior. Chen et al. (26) used the Susceptible-Exposed-Infected-Removed (SEIR) model to describe the state transition of individuals, studied the spread of public opinion in combination with the heterogeneity characteristics, such as individual herd, and verified the rationality and effectiveness of the model with the pricing event of the COVID-19 vaccine independently developed in China. Li et al. (27) integrated the particularity of panic buying public opinion, established a model of panic buying public opinion transmission, and analyzed the material panic buying problem caused by panic in an uncertain environment through computational simulation experiments. The above literature explains the reasons for the spread of panic buying behavior from the perspective of public psychology, and points out that social network media has become the main carrier of the rapid spread of panic. However, the above research has the problem of too much qualitative analysis and too little quantitative analysis.

It can be seen from the above analysis that current scholars explain the external causes of panic buying from the perspective of commodity supply and need balance, the internal causes of panic buying from the perspective of individual emotions, and the spread of panic buying from the perspective of public psychology. However, the perspective of supply and need only points out the impact of material need without considering the safety need that people should worry about while going out shopping, so it cannot fully fit the background of the epidemic. The perspective of the relationship between need and emotion is not identified in the thinking of emotional factors, and lacks quantitative research between emotion and behavior. There is also a lack of quantitative research on herd mentality.

Based on this, this paper uses the method of system dynamics to analyze the formation and dissemination process of panic buying behavior by introducing internal factors such as panic and individual needs, and external factors such as the influence of surrounding individuals and the change of external information. Combined with the SIR epidemic model, the whole process of panic buying behavior formation, disappearance, and recurrence is simulated, and the transmission model of panic buying behavior under the sudden epidemic situation is constructed. Then, with the help of computer simulation technology to simulate the whole process of the problem, we can understand the internal evolution mechanism of panic buying behavior, and analyze the impact of changes in real conditions.

## Methods

**Figure 1:**
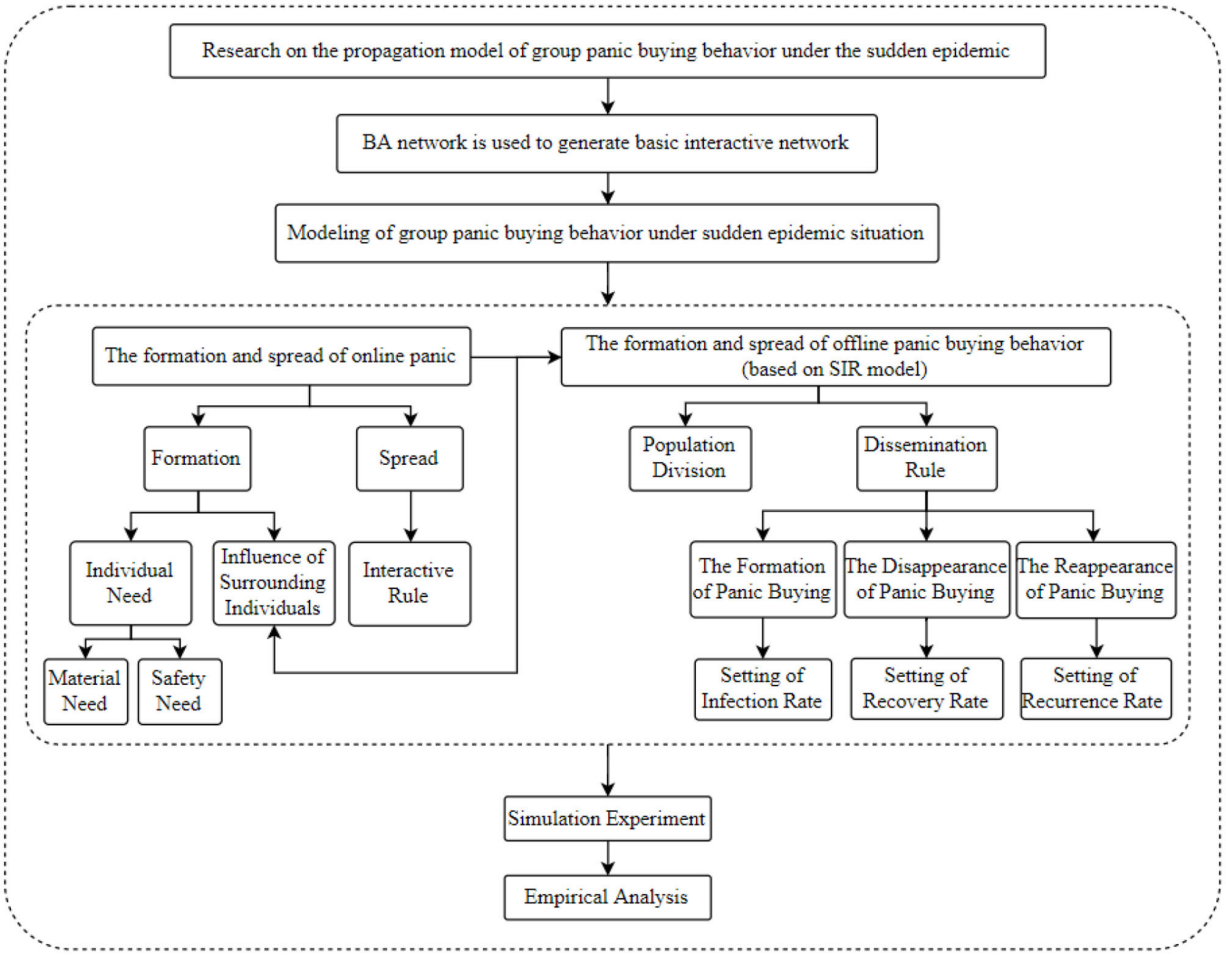
Research framework.

This paper is based on Monte Carlo's multi-agent method for modeling, using Agent to represent individual nodes in the network, and assuming that the network scale is *N*, that is, there are *N* netizen nodes in the network. The BA network is used as the basic network and the build panic buying propagation model is based on SIR model. The research framework of the paper is shown in Figure 1.

1. BA model
  BA model (28) refers to a scale-free network model, which was proposed by Albert-László Barabási and Réka Albert to explain the generation mechanism of the power law. BA model has two characteristics: the first is growth, which means that the network scale is increasing; the second is priority connection mechanism, which means that the new nodes in the network tend to connect with those nodes with a higher degree of connection. BA model can explain many phenomena, such as graduate students' choice of tutors. In this network, both graduate students and tutors are increasing, and graduate students always tend to choose tutors who have brought many graduate students.
2. SIR Model
  In the 1860's, Daley and Kendal found the similarities between infectious diseases and information transmission by comparing them. They first proposed the classic DK model (29), that is, the SIR model, which is the most widely used. In this model, the population is abstractly divided into three categories, susceptible, infected, and recovered individuals, corresponding to the individuals who do not know the information, the individuals who transmit the information, and the individuals who no longer participate in the information transmission. When individuals contact each other, there is a certain probability that they will transform each other. After that, scholars have carried out extended research on the basis of the SIR model, such as improving the crowd classification method, improving the propagation rules, and so on. For example, Chen et al. (26) added an individual category of exposed state and constructed the SEIR model. Exposed refers to the person who has been in contact with an infected person but has no ability to infect others.

**Figure 2:**
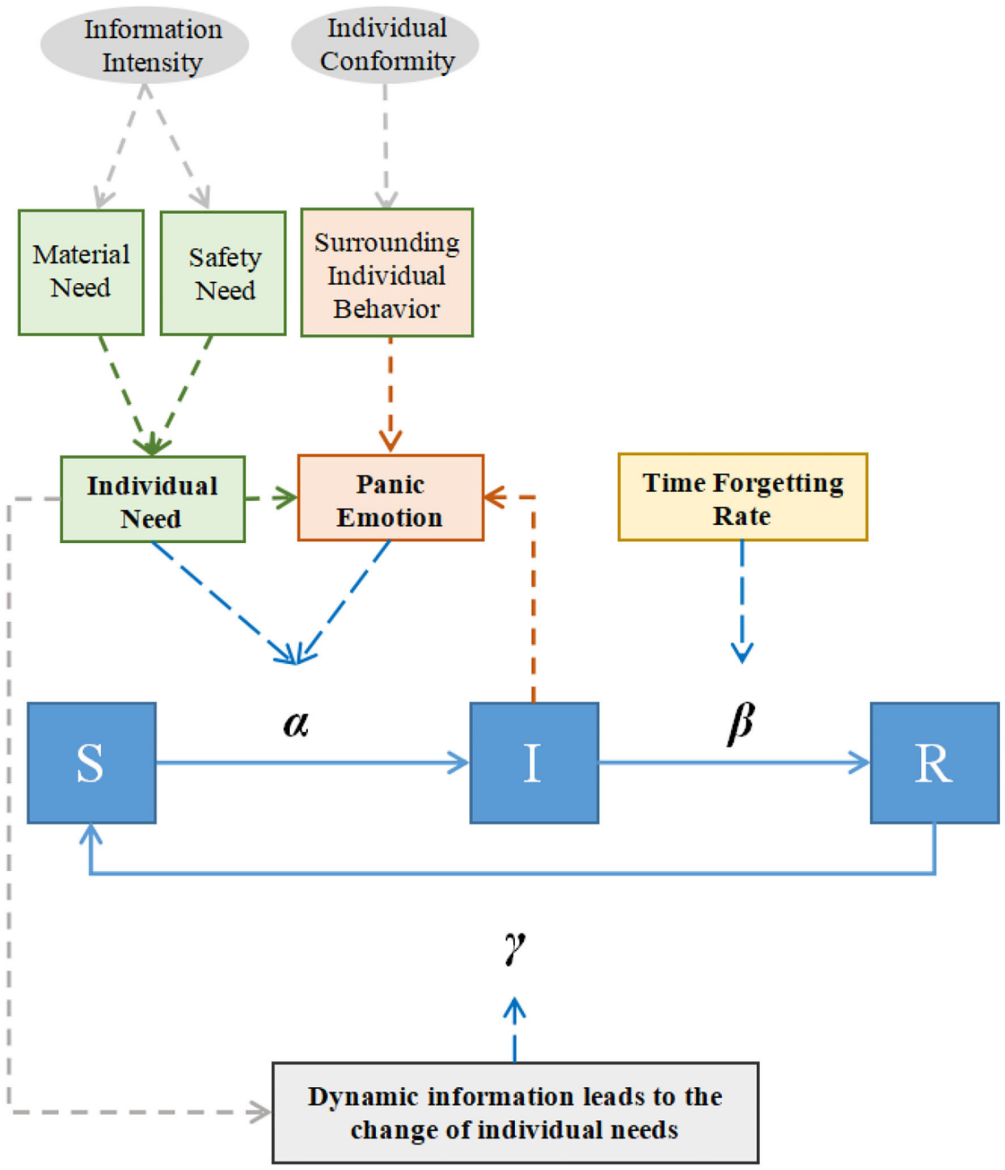
Model explanation.

Based on the SIR epidemic model, this paper constructs a panic buying propagation model, as shown in Figure 2. Under COVID-19, people learn about epidemic information through news and other methods. On the one hand, information such as shortages of supplies and the reappearance of the epidemic will cause people to panic. Under this influence, the individual transforms from a susceptible person (*S*) who never participates in panic buying into an infected person (*I*) who is a panic buyer with the probability α. On the other hand, the buying behavior of surrounding individuals will also cause panic among the people. The number of infected people (*I*) around the individual is used as an indicator to measure the influence of surrounding individuals, which further affects them. As the time goes by, the individual gradually forgets about it, and transforms from an infected person (*I*) into a recovered person (*R*) who is insensitive to the panic buying with probability β. Finally, when the relevant epidemic information is brought up again, the memory of the people is awakened again, and the recovered people (*R*) will transform into susceptible people (*S*) with probability γ, becoming panic buyers again. The parameters and variables involved in the model are shown in Table 1.

**Table 1:** Related parameters and variables.

*a*	The weight of material need (physiological need) in individual need
*b*	The weight of safety need in individual need
*Con*(*i*)	Conformity of individual *i*
μ_1_	Assimilation parameter
μ_2_	Exclusive parameter
*d*_1_	Assimilation threshold
*d*_2_	Exclusive threshold
*c*_1_	Parameter that affects the forgetting rate function for deciding the attraction of panic buying on the infected people
*c*_2_	Parameter that affects the forgetting rate function for deciding the shape of forgetting curve
*E_*i*_*(*t*)	Panic value of individual *i* at time *t*
*M_*i*_*(*t*)	Material need of individual *i* at time *t*
*S_*i*_*(*t*)	Safety need of individual *i* at time *t*
*F_*i*_*(*t*)	Influence of surrounding individuals on individual *i* at time *t*
*I_*M*+_*(*t*)	Intensity of positive information about material need
*I_*M*−_*(*t*)	Intensity of negative information about material need
*I_*S*+_*(*t*)	Intensity of positive information about safety need
*I_*S*−_*(*t*)	Intensity of negative information about safety need
*N_*i*_*(*t*)	Number of neighbor nodes around individual *i* at time *t*
*NI_*i*_*(*t*)	Number of neighbor nodes that take panic buying behavior around individual *i* at time *t*
*NS*(*t*)	Number of susceptible individuals *S* at time *t*
*NI*(*t*)	Number of infected individuals *I* at time *t*
*NR*(*t*)	Number of recovered individuals *R* at time *t*
*PS*(*t*)	Proportion of susceptible individuals *S* to all individuals at time *t*
*PI*(*t*)	Proportion of infected individuals *I* to all individuals at time *t*
*PR*(*t*)	Proportion of recovered individuals *R* to all individuals at time *t*
α	Infection rate
β	Recovery rate
γ	Recurrence rate
θ_1_	Influence weights of individual need on the buying behavior
θ_2_	Influence weights of panic on the buying behavior
*t*_1_	Duration of an individual becoming infected

### The Formation and Spread of Online Panic

Emotions are people's psychological feelings. In the process of behavior decision-making, various emotions will interfere with individual behavior judgments from a psychological level. Under the COVID-19 epidemic, panic is the most common emotion that interferes with individual behavior. It refers to a kind of depressive emotion that people may have while facing a certain dangerous situation. Under its effect, the individual's cognitive imbalance and the ability to make rational judgments are reduced, so they may perform various irrational behaviors. For example, during the period of COVID-19, facing the unpredictable future, people all over the world spontaneously panicked, triggering various panic buying events, such as the panic buying of hand sanitizer, toilet paper, and beverages in Canada and the United States, as well as rice in China. Under the influence of panic, people are more likely to be irrational and tend to conduct group behavior. At the same time, the predicament of forbidding going out has prompted people to confide their emotions more through online social networks, and the characteristics of no spatio-temporal limit, anonymity, and wide audiences of online social networks undoubtedly further promote the formation and spread of panic.

#### The Formation of Panic

The formation of online panic is affected by the individual's needs from the internal influence and from the external influence of surrounding individuals. All kinds of news related to the epidemic on the Internet can stimulate the actual needs of people. For example, seeing the news of other infected people enables individuals to perceive that they are in a dangerous environment and generate safety needs. The news of supply shortages will make individuals think about whether one's future life is guaranteed; in turn, there will be a need for supplies. If these actual needs are not met, the individual will panic. In addition, people will browse and publish information related to their lives on the Internet. Once they find their neighbors participate in panic buying, they will also have a buying desire due to group psychology. In reality, the unsafety of panic buying behavior increases their panic.

Based on the above analysis, *E*_*i*_(*t*) is used to represent the panic value of individual *i* at time *t* and *E*_*i*_(*t*)∈[0, 1]. The higher the value is, the higher the panic degree is. Its calculation formula is as follows:

(1) $$\begin{array}{l} E_{i}\left(\text{t}\right)={\text{a}}^{*}M_{i}\left(\text{t}\right)+{\text{b}}^{*}S_{i}\left(\text{t}\right)+F_{i}\left(\text{t}\right) \end{array}$$

where *a* and *b* are the weights of material need (physiological need) and safety need from an individual level, respectively (*a* + *b* = 1). Since physiological need is higher than safety need in Maslow's hierarchy of needs, set *a*>*b*; *M*_*i*_(*t*) and *S*_*i*_(*t*) are the material needs and safety needs of an individual *i* at time *t* respectively. *F*_*i*_(*t*) means the influence of surrounding individuals on individual *i* at time *t*.

##### Individual Need

Needs are the rational needs of social people, which also affect individual behavior. At present, the most typical need theory is Maslow's Hierarchy of Needs. The lowest and most prioritized need to be met is physiological need, which includes people's needs for food, water, air, and other basic materials. Only when people meet their physiological needs can they have a chance of survival. Secondly, safety needs correspond to people's uncertainty of the surrounding environment, natural uncertainty, and natural contradictions between people. During the period of COVID-19, people avoided going out as much as possible for the sake of safety. However, people had to find ways to purchase materials for the need of survival materials, so it might be necessary to go out. Therefore, from the perspective of individual needs, safety needs and physiological needs play a game with each other, which has an important influence on people's behavioral decisions, such as whether to go out or purchase goods during the epidemic period.

People's needs for supplies and safety will be affected by external information. During the epidemic, this information is mainly spread through online channels. After the information about materials and safety is received by people, everyone will synthesize the information they receive to form their own judgments on whether the external materials are sufficient and whether the external environment is safe, which are represented by material need *M*_*i*_(*t*) and safety need *S*_*i*_(*t*), respectively. The more abundant external materials are, the lower the material need is. The safer the external environment is, the lower the safety need is.

*Material need *M_*i*_*(*t*).* Material need *M*_*i*_(*t*) refers to the material need of individual *i* at time *t, M*_*i*_(*t*)∈(0, 1). The larger the value is, the higher the need for materials is, and the more people are prone to purchasing behavior. The calculation formula of the material need *M*_*i*_(*t*) is as follows:

(2) $$\begin{array}{l} M_{i}\left(t\right)=\frac{1-\left(I_{M+}\left(t\right)-I_{M-}\left(t\right)\right)}{2} \end{array}$$

where *I*_*M*+_(*t*) is the intensity of positive information about material need, and *I*_*M*−_(*t*) is the intensity of negative information about material need. If the intensity of positive information is stronger and the intensity of negative information is weaker, it means that the material is sufficient and the need for material is lower. In actuality, the intensity of information can be measured by the number of readings, page views, and likes of the information on platforms such as Twitter, Facebook, and Sina Weibo. Generally speaking, *I*_*M*+_(*t*)∈(0, 1), *I*_*M*−_(*t*)∈(0, 1).

*Safety need *S_*i*_*(*t*).* Similarly, with regard to the safety needs *S*_*i*_(*t*)∈(0, 1), the higher the value is, the higher the vigilance of the individual to the external environment is, and the more insecure the external environment is, the less easy it is to go out. The calculation formula of safety need *S*_*i*_(*t*) is as follows:

(3) $$\begin{array}{l} S_{i}\left(t\right)=\frac{1-\left(I_{S+}\left(t\right)-I_{S-}\left(t\right)\right)}{2} \end{array}$$

where *I*_*S*+_(*t*) is the intensity of positive information about safety need, and *I*_*S*−_(*t*) is the intensity of negative information about safety need. If the intensity of positive information is stronger and the intensity of negative information is weaker, it means that the safety is higher and the safety need is lower. In general, *I*_*S*+_(*t*)∈(0, 1), *I*_*S*−_(*t*)∈(0, 1).

##### Influence of Surrounding Individuals

People, as part of a social group, are influenced by the individuals around them. During the period of COVID-19, when relatives and friends released pictures of buying goods or netizens published tips for buying goods on social media, these buying behaviors would cause uneasiness and anxiety, making people more uncertain about whether to go out to purchase goods in such a dangerous environment, and further stimulating the formation of people panic.

The influence of the surrounding individuals is defined as *F*_*i*_(*t*), which is related to the number of people around the individuals who take panic buying behavior and the conformity of the individuals. The calculation formula is as follows:

(4) $$\begin{array}{l} F_{i}\left(t\right)=\frac{NI_{i}\left(t\right)}{N_{i}\left(t\right)}*Con\left(i\right) \end{array}$$

where *N*_*i*_(*t*) represents the number of neighbor nodes around individual *i* at time *t*, and *NI*_*i*_(*t*) represents the number of neighbor nodes that take panic buying behavior around individual *i* at time *t*. The more neighbor nodes are around the individual that take panic buying behavior, the easier it is to trigger panic. *Con*(*i*) represents the conformity of individual *i*, which is related to social factors such as the growth environment and educational background of the individual.

#### The Spread of Panic

Panicked people usually feel uneasy and anxious. Many people choose to vent their negative emotions on online social networks, such as Twitter, Facebook, and Sina Weibo, while others who follow their accounts see this may be affected, which affects more people. As a result, panic is further spread in online social networks.

The J-A model proposed by Jager and Amblard (30) is an important model of opinion interaction, which considers assimilation, repulsion, and neutrality in social evaluation theory. Based on this, a panic spread model is established. Assuming that individuals *i* and *j* interact, the interaction rules are as follows:

1. If the emotion values of individuals *i* and *j* are similar, the psychology of convergence will occur, and the emotion value will be closer.
2. If the emotion value differences between individuals *i* and *j* are large, rebellious psychology will occur, and the difference will increase.
3. In other cases, the emotions of the two individuals remain unchanged.

According to the interaction rules, the emotion values of the individuals *i* and *j* after the interaction are updated. The calculation formula is as follows:

(5) $$\begin{array}{l} \begin{array}{cc} E_{i}(t)= & \left\{ \begin{array}{l} \begin{array}{ccc} \begin{array}{l} E_{i}(t)-\mu_{1}*\left(E_{i}(t)-E_{j}(t)\right)\;,\text{if}\\ E_{i}(t)-\mu_{2}*\left(E_{i}(t)-E_{j}(t)\right)\;,\text{if} \end{array} & \left|\begin{array}{l} E_{i}(t)-E_{j}(t)\\ E_{i}(t)-E_{j}(t) \end{array}\right| & \begin{array}{l} <d_{1}\\ >d_{2} \end{array} \end{array}\\ E_{i}(t)\;,\text{others} \end{array}\right. \end{array}\\ \begin{array}{cc} E_{j}(t)= & \left\{ \begin{array}{l} \begin{array}{ccc} \begin{array}{l} E_{j}(t)-\mu_{1}*\left(E_{j}(t)-E_{i}(t)\right)\;,\text{if}\\ E_{j}(t)-\mu_{2}*\left(E_{j}(t)-E_{i}(t)\right)\;,\text{if} \end{array} & \left|\begin{array}{l} E_{j}(t)-E_{i}(t)\\ E_{j}(t)-E_{i}(t) \end{array}\right| & \begin{array}{l} <d_{1}\\ >d_{2} \end{array} \end{array}\\ E_{j}(t)\;,\text{others} \end{array}\right. \end{array} \end{array}$$

where μ_1_ is the assimilation parameter, μ_2_ is the exclusive parameter, *d*_1_ is the assimilation threshold, and *d*_2_ is the exclusive threshold.

### The Formation and Spread of Offline Buying Behavior

From a micro perspective, the behavior of individuals participating in offline panic buying is comprehensively affected by factors such as panic and individual needs. The higher the individual material need is, the lower the safety need is and the higher the panic is, the easier it is to promote individual participation in panic buying. From a macro perspective, probability is adopted to measure the occurrence of individual panic buying behavior, and the SIR model is used to build the spread process of offline panic buying behavior.

#### Group Division

According to the principle of the SIR infectious disease model, the group is divided into three categories - *S*(Susceptible people), *I*(Infected people), and *R*(Recovered people) - with panic buying behavior as the content of spread. Susceptible people refers to people who are not panic buying but are easily affected. Infected people refers to people who are currently engaged in panic buying. Recovered people refers to people who have participated in the panic buying but are not involved now.

**Figure 3:**
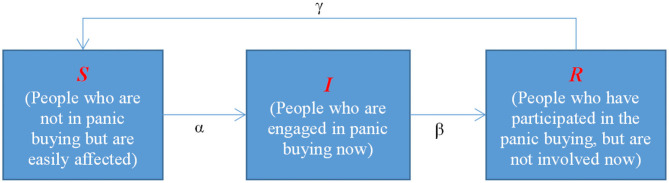
Transformation of the relationship among three categories.

At time t, the number of susceptible, infected, and recovered individuals are recorded as *NS*(*t*), *NI*(*t*), *NR*(*t*), and their proportions to all individuals are *PS*(*t*), *PI*(*t*), *PR*(*t*), obviously *PS*(*t*)+*PI*(*t*)+*PR*(*t*) = 1. Supposing *PS*(*t*), *PI*(*t*), and *PR*(*t*) are continuous and differentiable function about time *t*, the initial proportions of three kinds are defined as *NS*(0), *NI*(0), and *NR*(0). The transformation of the relationship among *S, I*, and *R* is shown in Figure 3.

#### Spread Rules

As shown in Figure 3, the spread rules for panic buying are as follows:

① The formation of panic buying behavior: Under the comprehensive influence of individual needs and panic, the susceptible people (*S*) are transformed into infected people (*I*) at the infection rate α.
② The disappearance of panic buying behavior: As time goes by, individuals will gradually forget about this. The infected people (*I*) are transformed to the recovered people (*R*) at the recovered rate β.
③ Reappearance of panic buying behavior: When the relevant epidemic information is brought up again, the people's memory is awakened again, and the people who are recovered people (*R*) are transformed into susceptible people (*S*) at the recurrence rate γ.
④ Repeat the above steps.

The differential equation of SIR model is shown in Equation (6) as follows:

(6) $$\begin{array}{l} \left\{ \begin{array}{c} \frac{dS}{dt}=\gamma RS-\alpha SI\\ \frac{dI}{dt}=\alpha SI-\beta IR\\ \frac{dR}{dt}=\beta IR-\gamma RS\\ N=S+I+R \end{array}\right. \end{array}$$

where α is the infection rate, and the individual infection process is expressed as SI. β is the recovered rate, and the process from the infected to the recovered is expressed as IR. γ is the recurrence rate, and the process of recovered people to susceptible people is expressed as RS.

##### Setting of Infection Rate α

The infection rate α is related with individual needs and panic. The higher the material need *M*_*i*_(*t*) is, the more individuals have the desire to buy. The lower the *S*_*i*_(*t*) is, the more confident individuals are to go out. The higher the panic value of *E*_*i*_(*t*) is, the easier it is for individuals to abandon rational thinking and adopt panic buying behavior. Therefore, the calculation formula of infection rate α can be expressed as follows:

(7) $$\begin{array}{l} \alpha =\theta_{1}*\left(a*M_{i}\left(t\right)+b*\left(1-S_{i}\left(t\right)\right)\right)+\theta_{2}*E_{i}\left(t\right) \end{array}$$

where θ_1_ and θ_2_ are the influence weights of individual need and panic on the buying behavior. *a* and *b* are the weights of the physiological need *M*_*i*_(*t*) and safety need *S*_*i*_(*t*) in individual needs, *a* + *b* = 1 and a > b.

The higher the value of panic is, the more irrational the individual is, the stronger the effect of emotion is on individual buying behavior, and the weaker the effect of individual need is on buying behavior. Therefore, the value of panic can be used to measure the influence weight, as follows:

(8) $$\begin{array}{l} \left\{ \begin{array}{c} \theta_{1}=|1-E_{i}\left(t\right)|\\ \theta_{2}=|E_{i}\left(t\right)| \end{array}\right. \end{array}$$

Since the formation of panic is related to the surrounding individuals, as time goes by, when the panic continues to spread or there are more and more surrounding individuals to buy things, the infection rate of individuals will further increase, thus forming the spread of buying behaviors.

##### Setting of Recovery Rate β

When an individual becomes susceptible *S* and is in a state of panic buying behavior, if there is no new and dynamic epidemic information, the longer the time passes, the more the individual will forget this and no longer participate in the panic buying. Nekovee et al. (31) introduced the forgetting mechanism when studying the rumor propagation model. Therefore, the recovery rate β is related to time. Referring to Nekovee's literature, the specific calculation formula is as follows:

(9) $$\begin{array}{l} \beta =c_{1}-e^{-c_{2}\cdot t_{1}} \end{array}$$

where *c*_1_ and *c*_2_ are the parameters of the forgetting probability function, and *t*_1_ represents the duration of an individual becoming infected. When *t* = 0, β = *c*_1_−*1*, that is, the forgetting rate at the initial moment is *c*_1_−1, representing the initial attraction of the buying behavior to the infected people. The parameter *c*_2_ determines the shape of the forgetting curve. The larger the value is, the faster the forgetting rate changes and the easier it is to forget.

##### Setting of Recurrence Rate γ

When there is a new outbreak, all kinds of epidemic information reappear in the public, stimulating recovered people (*R)* to be panic buyers again and transform to susceptible people (*S*), starting the next round of panic buying. For example, when the epidemic reached the United Kingdom in March 2020, many places witnessed panic buying; hand sanitizer, toilet paper, and other daily supplies were out of stock. After the release of the new epidemic blockade measures in the UK in December, people feared that there were not enough Christmas supplies. Supermarkets in London, Cardiff, Newcastle, and other places witnessed “frantic panic buying.” Therefore, the calculation formula of recurrence rate γ is as follows:

(10) $$\begin{array}{l} \gamma =\left\{ \begin{array}{c} \frac{M_{i}\left(t\right)+\left(1-S_{i}\left(t\right)\right)}{\text{2}},\text{if }M_{i}\left(t\right)>M_{i}\left(t-1\right)\text{ or }S_{i}\left(t\right)<S_{i}\left(t-1\right)\;\\ 0,\text{others} \end{array}\right. \end{array}$$

When *M*_*i*_(*t*) increases or *S*_*i*_(*t*) decreases, it means that the negative information about materials increases, and the positive information about safety increases, which will stimulate the individual to purchase outside. In other cases, the recurrence rate is 0.

**Figure 4:**
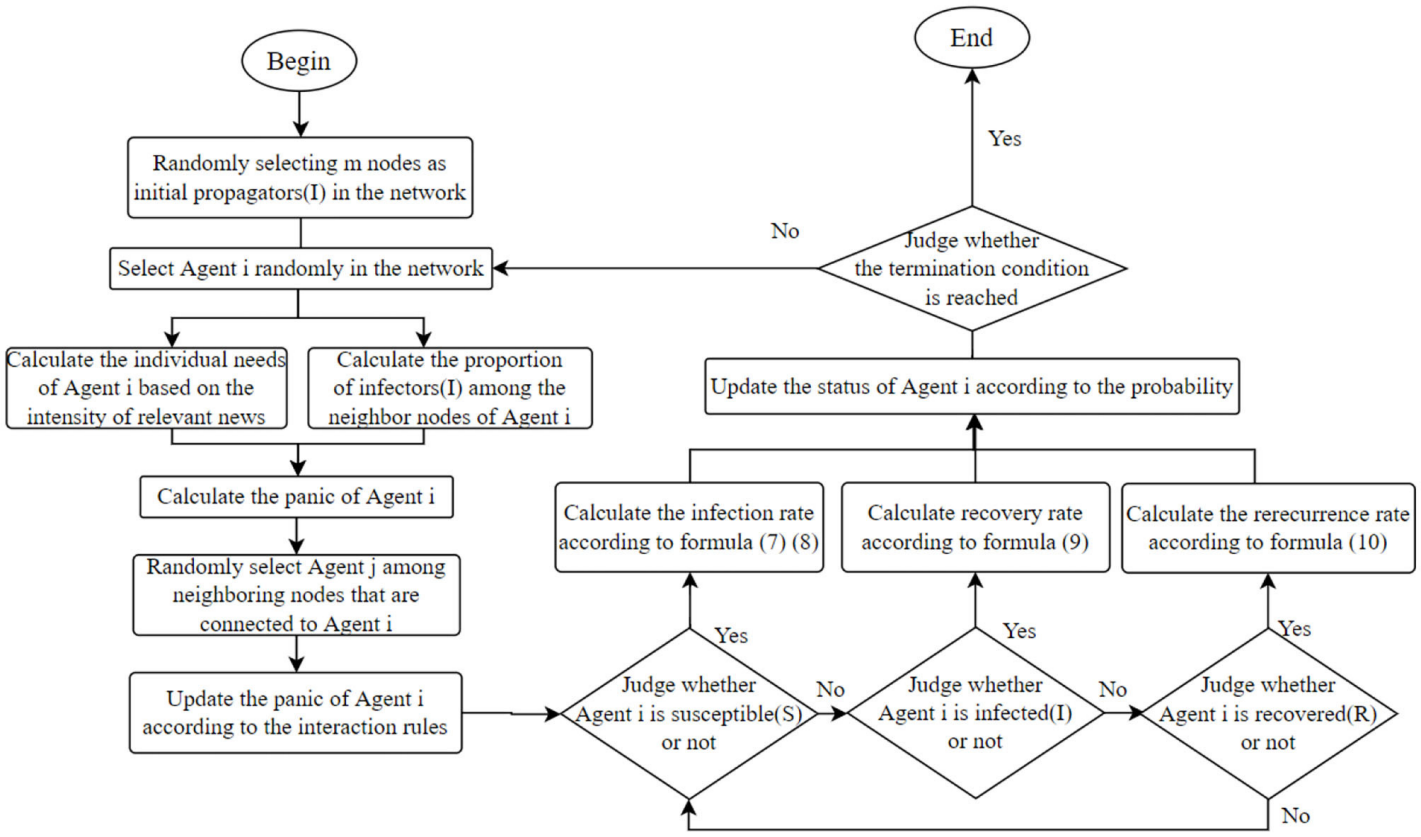
Evolution process.

Based on the above analysis, the evolution process of panic buying behavior under the sudden epidemic is shown in Figure 4.

## Results

In this section, MATLAB is used to simulate the model constructed above, analyzing the influence of individual needs, panic, individual conformity, interaction times, and the released time of external information on panic buying behavior to reveal its internal evolution mechanism.

BA scale-free network is selected as the initial network for the simulation experiment, and the node size is 1,000. According to Maslow's hierarchy theory of needs, physiological needs are more basic and more important than safety needs. Therefore, set *a* = 0.6 and *b* = 0.4. According to the central limit theorem, people's height, shoe size, surrounding environment, and so on are subject to normal distribution. Therefore, the individual conformity degree *Con*(*i*) is set to follow the normal distribution of N~(0.5,0.15), the value >1 is set as 1, and the value <0 is set as 0, so that the parameter is mapped within the interval of [0, 1]. The mean value of 0.5 indicates that most individuals in the group are in the middle of conformity, and the variance of 0.15 is to make all the numbers within the range of [0, 1] reach the probability value. Comprehensive visualization consideration, the proportion of individuals (i.e., infected people *I*) who participate in panic buying at the initial moment is set to be 6%, and the remaining individuals are susceptible people. The parameters of J-A model are set as μ_1_ = 0.2, μ_2_ = 0.2, *d*_1_ = 0.2, *d*_2_ = 0.6. The parameters of forgetting probability function in immunity rate are set as *c*_1_ = 1, *c*_2_ = 0.01.

### The Influence of Individual Needs and Panic on the Spread of Panic Buying

**Figure 5:**
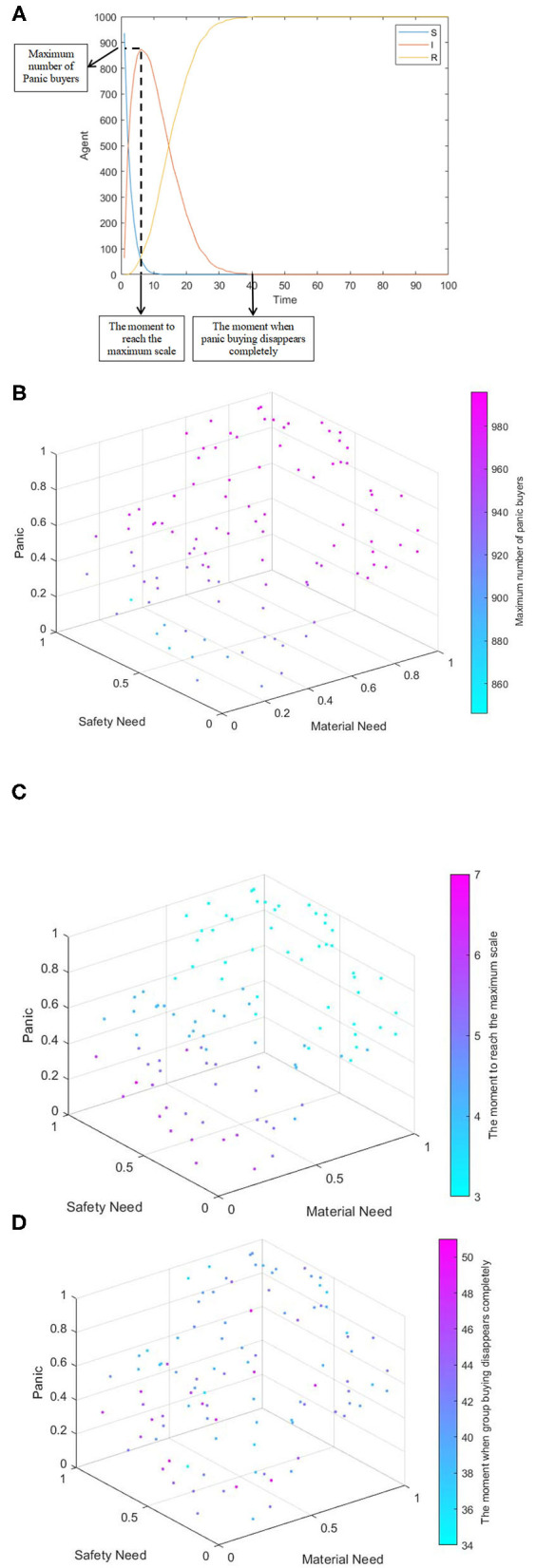
The comprehensive influence of individual need and panic on the spread of panic buying. **(A)** Indications demonstration. **(B)** Four-dimensional scatter diagram of safety need, material need, panic, and maximum number of panic buyers. **(C)** Four-dimensional scatter diagram of safety need, material need, panic emotion, and the moment to reach the maximum scale. **(D)** Four-dimensional scatter diagram of safety need, material need, panic emotion, and the moment when panic buying disappears completely.

Individual needs and panic are the direct factors affecting buying behavior. Due to the long-term inability to go out during the epidemic, in order to prevent shortage of supplies and meet their own physiological needs, people may rush to buy, hoarding a large amount of supplies at once. In addition, under the influence of panic, people with sufficient supplies may follow others and participate in panic buying. At the same time, there is a certain correlation between individual needs and panic. Individual needs are the internal factors causing panic. In order to analyze the influence of individual needs (material need, safety need) and the panic on the spread of panic buying behavior, different individual needs are set by random distribution. One hundred simulation experiments were carried out and the following information was recorded: the initial average material need, the initial average safety need, the initial panic value, maximum number of panic buyers, the moment to reach the maximum scale, and the moment when panic buying disappears completely. The demonstration of the maximum number of panic buyers, the moment to reach the maximum scale, and the moment when panic buying disappears completely is shown as Figure 5A.

Figure 5 shows the comprehensive influence of individual demand and panic on panic buying. As can be seen from Figure 5B, the lower the safety need is, the higher the material need is, the higher the panic is, the more the maximum number of panic buyers is, and the larger the scale of panic buying is. As can be seen from Figure 5C, the higher the safety need is, the lower the material need is and the lower the panic is, so the shorter the time to reach the maximum scale of panic buying is. Moreover, the change brought by material need is greater than the change brought by safety need, indicating that the moment to reach the maximum scale of buying is more affected by the change of material need. As can be seen from Figure 5D, the moment when panic buying disappears completely has little correlation with individual needs and panic. Therefore, material need and panic have a positive impact on the scale and moment of panic buying, which has a negative impact on the scale of panic buying and the moment to reach the maximum scale. Individual need and panic have no obvious correlation with the disappearance of panic buying.

### The Influence of Individual Conformity on the Spread of Panic Buying

People are always influenced by the information around them. Conformity measures the degree to which individuals are influenced by those around them. In general, the greater the conformity is, the greater the influence will be. The following three different conformity degrees are set to compare the influence of individual conformity on the spread of panic buying behavior.

**Figure 6:**
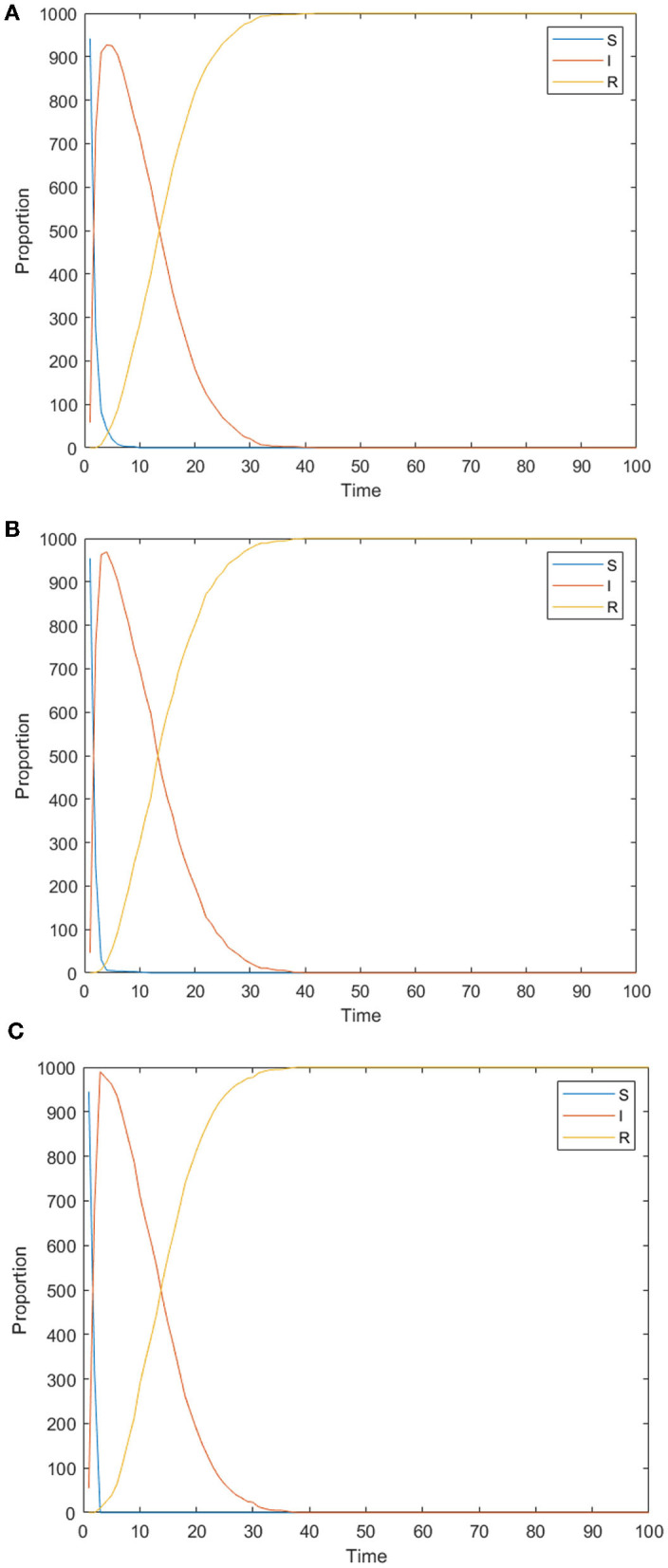
changes of panic buyers over time under different conformity degrees. **(A)** *Con*(*i*) obeys N~(0.2, 0.15). **(B)** *Con*(*i*) obeys N~(0.5, 0.15). **(C)** *Con*(*i*) obeys N~(0.8, 0.15).

Figure 6 shows the changes of panic buyers over time under different conformity degrees. Figures 6A–C shows the situations where the conformity degree *Con*(*i*) obeys N~(0.2, 0.15), N~(0.5, 0.15), and N~(0.8,0.15) respectively, simulating the situation that the individual conformity degree in the network is generally low, medium, and high. As can be seen from the figure, the higher individual conformity indicates more panic buyers and larger buying scale. It may be affected by the effect of conformity on panic. With a higher degree of panic among people, more people will participate in panic buying. Through communication with people, individuals will also feel panic and want to participate in the panic buying. To verify this idea, the influence of individual conformity on panic emotion is further analyzed below.

**Figure 7:**
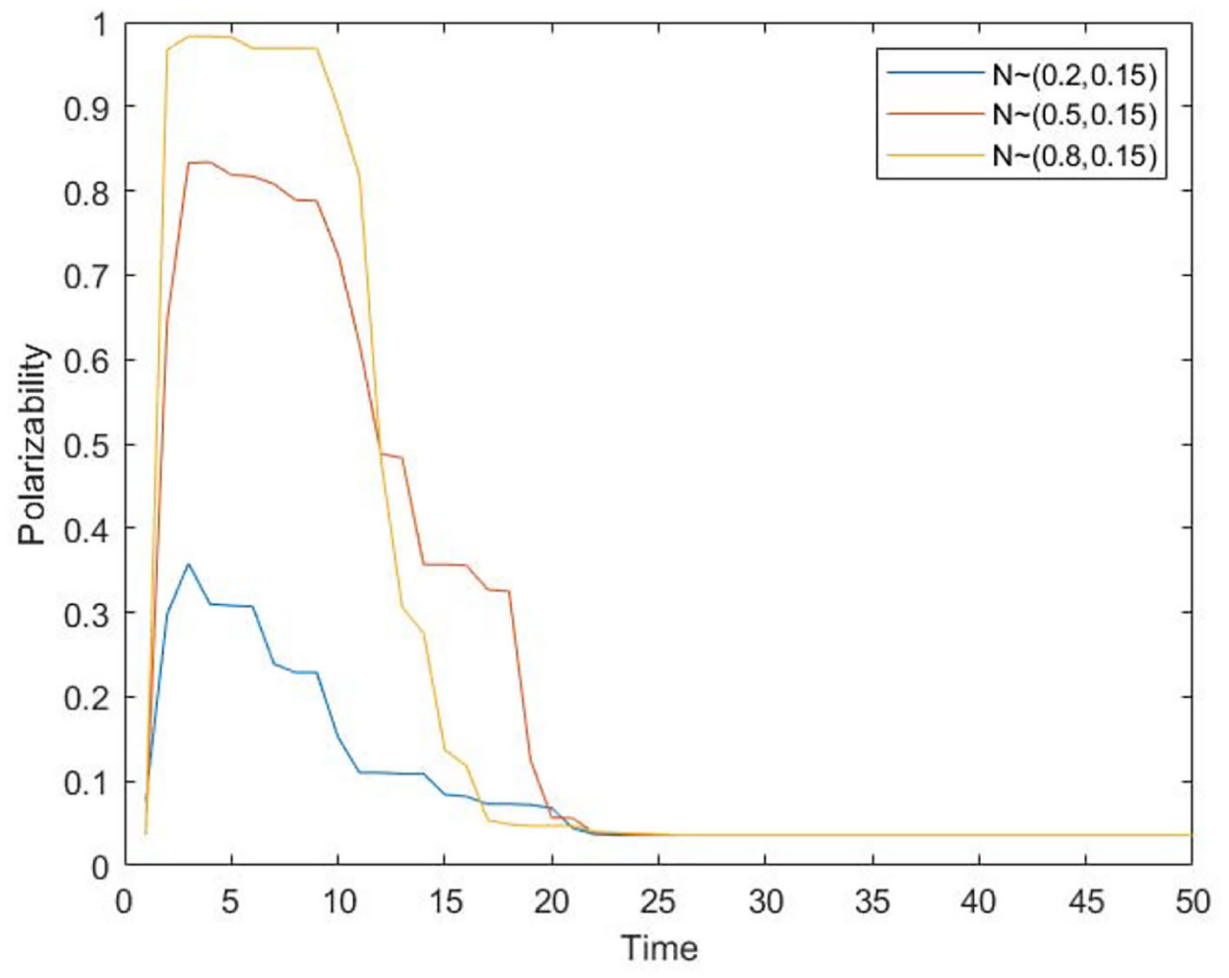
The change of panic polarizability over time under different conformity degrees.

We take all individuals in the group as the unit to observe the change of group panic through the polarization rate of panic. Assuming that the panic emotion value of 0.9 and above is extreme panic, the proportion of individuals with extreme panic in the whole is recorded as panic polarizability, and the polarizability under different conformity degrees is recorded. The results are shown in Figure 7.

As can be seen from Figure 7, the polarization of panic emotion first rises and then falls. The rise of the curve represents the increase of group panic. The initial panic buying occurs and is influenced by other individuals. The individual panic spreads continuously, leading to the rise of polarization. In the latter stages, the panic buying has dissipated and the panic has abated. This is because in the early stage of spreading, the higher the individual conformity is, the higher the polarization rate of panic (the wider spreading range) and the shorter the time to reach the highest polarization rate (the faster spreading speed) will be, which is consistent with the conclusion in Figure 6. In addition, in the later stage of spreading, the higher the individual conformity is, the faster the rate of panic polarization decreases. Higher conformity means listening to the opinions of others is easier, and the panic will dissipate faster. Therefore, although the increase of conformity makes the panic spread more widely and spread faster, it also makes the panic dissipate faster. In real life, in order to alleviate people's panic, relevant departments can guide people not to follow blindly and maintain independent thinking ability in the early stage of the event. In the later stages of the event, people can be guided to listen to others.

### The Influence of Different Connection Numbers on the Spread of Panic Buying

**Figure 8:**
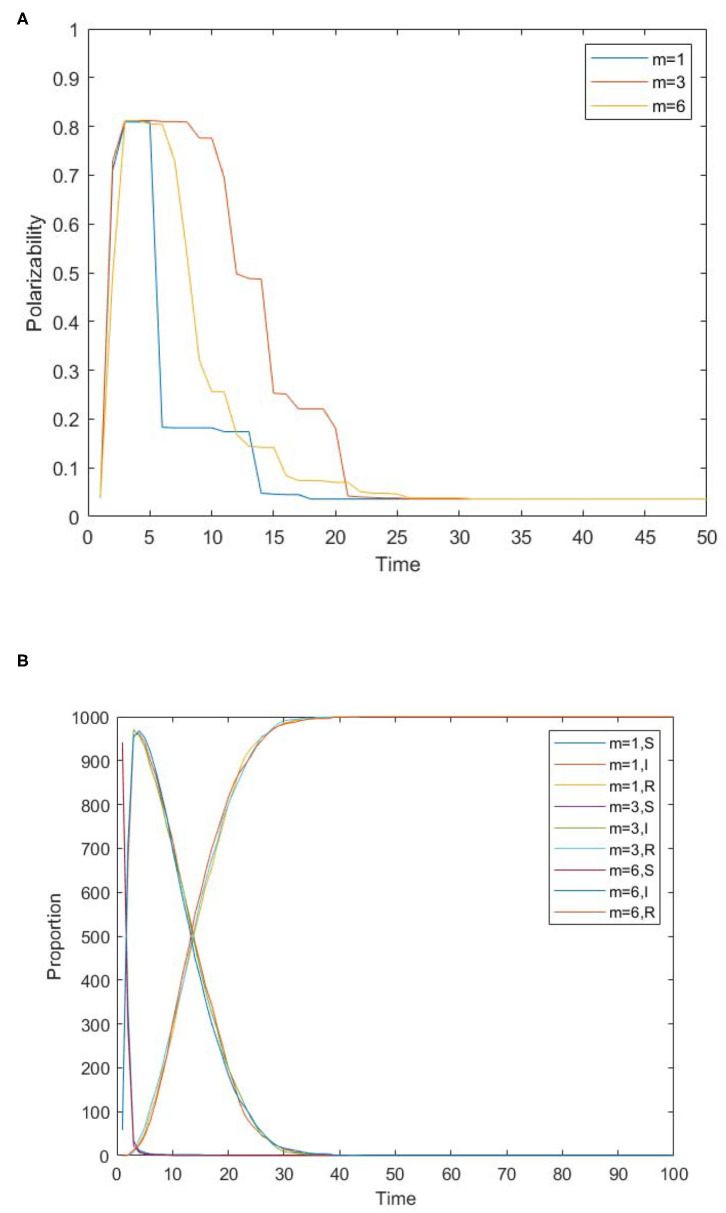
The influence of interaction number on the spread of panic buying. **(A)** The change of panic polarizability over time under different numbers of connections. **(B)** The distribution of panic buyers over time under different numbers of connected edges.

In the analysis in the previous section, individual conformity will affect the spread of panic and then panic buying behavior. However, if the individual has a smaller social circle and fewer people to communicate with, will the spread of panic and panic buying behavior be affected? Therefore, BA scale-free networks with different connection numbers are set up. BA scale-free network is a network generated according to the adoption of growth mechanism and priority connection mechanism. It changes the number of edges *m* increased each time, so as to understand the influence of interaction number on the spread of panic buying behavior. The simulation results are shown in Figure 8.

Figure 8 shows the influence of interaction number on the spread of panic buying. Figure 8A shows the change of panic polarizability over time under different numbers of connections, which show different effects at different stages. Time∈(1, 6) is the formation stage of panic emotion. At this time, the number of connections has no obvious effect on panic. Time∈(6, 20) is the main stage of panic remission. At this time, the panic polarizability is the highest when *m* = 3, followed by *m* = 6, and finally *m* = 1. This shows that the number of communicators does not faster reduce panic emotion. Instead, there is a threshold. When the number of nodes in the network reaches this threshold, the panic will reduce the fastest; over or under this threshold, the rate will slow down. In real life, if there are too many communicators, the individual may need to consider more and be more cautious. If there are too few communicators, they may be more self-centered and opinionated. Time∈[20, 50] is the final stage of the panic reduction. At this time, the reducing speed of the panic is proportional to the number of node edges. In the case of *m* = 1, the equilibrium is reached at Time = 20. In the case of *m* = 3, a balance is reached at Time = 22. In the case of *m* = 6, a balance is reached at Time = 26. Therefore, in order to alleviate the panic among the people, the relevant departments must distinguish the stages. In the initial stage of panic reduction, their best strategy is to properly grasp the connection between people, and not to deliberately block or guide people communicate. When the panic has dropped to a certain level, the communication among members of the society should be reduced as much as possible, and the channels of information spread should be reduced.

Figure 8B shows the distribution of panic buyers over time under different numbers of connected edges. It can be seen from the figure that the number of edges has no influence on the spread of panic buying behavior, so the number of people interacting has little influence on the spread of panic buying behavior. On the one hand, it may be that the influence of interaction number on panic emotion is mainly in the withdrawal period of emotion, and has no influence on the formation of panic emotion in the early stage, so there is little correlation on the spread of panic buying behavior. On the other hand, it may be because there are fewer isolated nodes in the network. Although there are differences in the number of individuals interacting with each other, it is still a closely connected network in general, and there is no isolated small group, therefore, it is easy to interact with each other, resulting in a chain reaction and forming panic buying.

### The Influence of the Released Time of External Information on the Spread of Panic Buying

**Figure 9:**
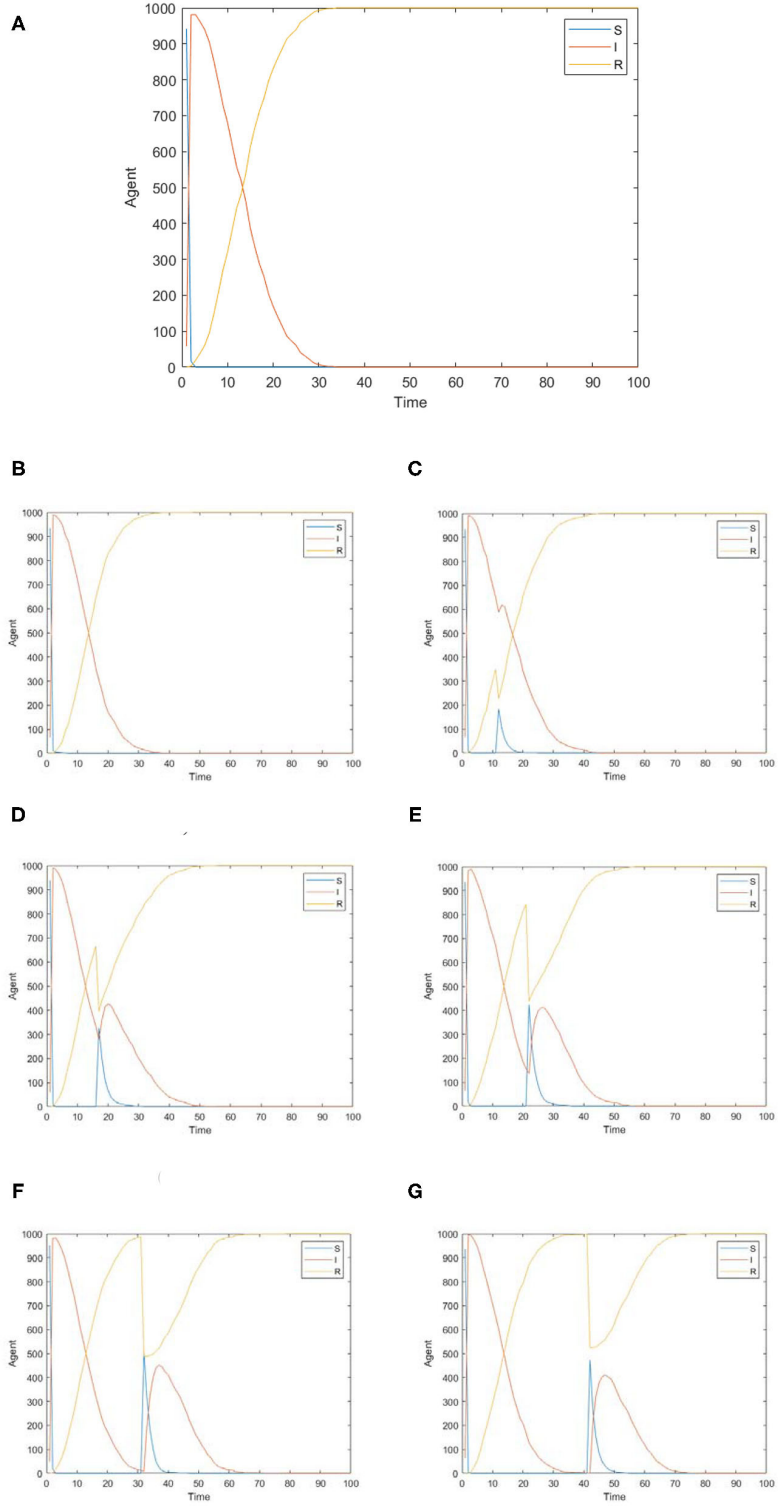
The distribution of panic buyers over time under different release times of external information. **(A)** The epidemic information unchanged. **(B)** *t* = 2. **(C)** *t* = 10. **(D)** *t* = 15. **(E)** *t* = 20. **(F)** *t* = 30. **(G)** *t* = 40.

Changes in external information will lead to changes in people's needs, which will affect people's desire to buy. For example, Chen et al. (32) studied the polarization of multi-dimensional public opinion and they found that the intervention of external information in different times and dimensions will affect the spread of public opinion. Keane and Neal (33) constructed a daily consumer panic index for 54 countries from January to April 2020. Research shows that the announcement of movement restrictions at the beginning of a pandemic can cause more panic than later announcements. Thus, this section will analyze the impact of the released time of external information on the spread of panic buying behavior, which will help relevant departments to explore the best time to release information, so as to better grasp the opportunity for intervening. The situation where the epidemic information about safety and supplies is negative and unchanged is studied first. The simulation results are shown in Figure 9A. It can be found that the number of infected people (*I*) reaches the maximum at *t* = 3, and the curves of infected people (*I*) and recovered people (*R*) intersect at *t* = 15. At *t* = 32, the number of infected people (*I*) drops to 0, and no individuals are participating in panic buying. Later, the influence of changes in external information on the spread of panic buying is observed, and the general direction of the change of epidemic information is that the material-related information changes from negative to positive, and the safety-related information changes from negative to positive, that is, material need and safety need are gradually reduced. Taking the time *t* = 3, 15, and 32 as anchor points, through the adjustment of the time points of the epidemic information change, the changes in the spread of panic buying are discussed. The change time points of the epidemic information are set to *t* = 2, 10, 15, 20, 30, and 40, respectively. The results are shown in Figures 9B–G.

Figures 9B–G shows the changes at *t* = 2, 10, 15, 20, 30, and 40. Comparing Figures 9B,C and Figure 9A, we can see that if the epidemic information changes before the first panic buying reaches a maximized scale, it will not cause a second wave of panic buying, otherwise there will be. Comparing Figures 9A,C–E, it can be seen that if the epidemic information changes occur before the *I* and *R* curves intersect, the impact on panic buying will fluctuate less, and vice versa. When *t* = 30, the first panic buying is about to end, and when *t* = 40, the first panic buying has completely ended. Comparing Figures 9A,F,G, it can be seen that the maximum size of the second panic buying at *t* = 30 is about 450 people, and the maximum size of the second panic buying at *t* = 40 is about 400 people. This shows that when the first panic buying is about to end, if the scale of the second wave of panic buying is to be controlled, the effect of releasing positive information after the first panic buying is better than before the end.

## Discussion

This paper selected panic buying cases in China and the United Kingdom, and verified the panic buying model through text analysis and simulation modeling based on real data.

### Case Analysis

#### Case 1: Panic Buying in Shijiazhuang, China

Since December 2019, some hospitals in Wuhan, Hubei Province, China initially discovered multiple cases of pneumonia of unknown cause. Subsequently, this virus spread rapidly around the world. In February 2020, Tedros Adhanom Ghebreyesus, the Director-General of the World Health Organization, announced that the pneumonia caused by the new coronavirus was named “COVID-19.” In China, as a result of positive public health intervention measures, various provinces have resumed work and production from March 2020, and universities have begun to organize resumption of school from April 2020, and social life has basically returned to normal (34).

However, on January 4, 2021, 127 COVID-19 infected people reappeared in Shijiazhuang, Hebei Province, China. Shijiazhuang government urgently declared the need to enter a state of war (35). On January 6, 2021, citizens of Shijiazhuang rushed to the supermarket to buy daily necessities such as rice, noodles, grains, and oil (36). From January 7th to January 10th, in order to avoid another panic buying boom, the Shijiazhuang government released news about the guaranteed basic living materials when releasing information about the epidemic. For example, 70 supermarkets in Shijiazhuang promised not to increase the price of storage-resistant vegetables (37). These topics are widely discussed by netizens on Sina Weibo.

Sina Weibo is China's leading social media Weibo company. It has interactive functions such as follow, like, comment, and forward. Currently, Sina Weibo has more than 511 million monthly active users, and a large number of netizens' comments on various events have been accumulated on the platform. Therefore, this paper takes Sina Weibo as the case data source.

**Figure 10:**
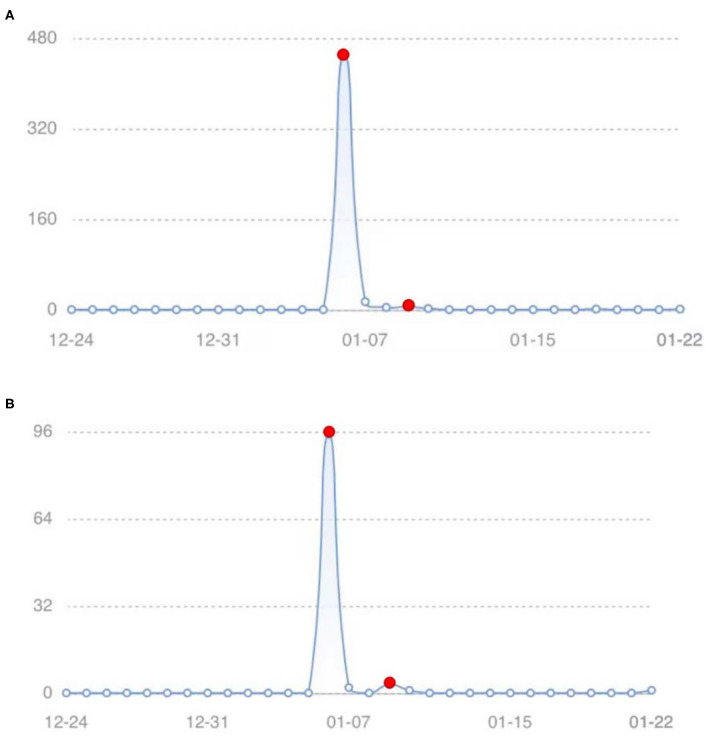
Sina weibo topic index trend of #Shijiazhuang residents rush to buy rice, flour, grain, and oil# (data from Sina Weibo). **(A)** Discuss trend. **(B)** Original trend.

Figure 10 is a Sina Weibo topic index trend with regard to #Shijiazhuang residents rush to buy rice, flour, grain, and oil#. The trend represents the change in the number of netizens who publish related Weibo content by themselves, as well as comments, likes, and reposts related to others' Weibo content. The original trend represents the change in the number of related Weibo content that netizens publish by themselves, which can reflect the enthusiasm of Shijiazhuang panic buyers to a certain extent. There are two crests in the two trend graphs (indicated by red dots in the figure), the big crest on January 6 and the small crest on January 9, which shows that the panic buyers have shown a two-stage change in panic buying. The enthusiasm of netizens for panic buying in the first stage is very high, while the enthusiasm of netizens for panic buying in the second stage is very low.

In order to study the panic buying behavior of “Shijiazhuang residents rush to buy rice, flour, grain, and oil,” this section cites the data analysis method of Chen et al. (38) to analyze the comments of Sina Weibo topic of #Shijiazhuang residents rush to buy rice, flour, grain, and oil#. We divided the event into two stages, focusing on the time when the topic first appeared: the first stage is the first panic buying period, that is, from January 4, 2021 to January 6, 2021, and the second stage is the period when the positive information about the materials is released after the first panic buying, that is, from January 7, 2021 to January 10th, 2021. On Sina Weibo, news topics related to the safety and supplies of the Shijiazhuang epidemic were crawled in two time periods. A total of 11 Weibo topics and 17,856 Weibo comment data under the topics are crawled. The topic division is shown in Table 2. Although the amount of data obtained here is limited, according to the six-degree separation theory in interpersonal relationships, the statistical results of these user data can reflect the general applicability of Weibo user behavior to a large extent.

**Table 2:** Topic division.

**Time**	**News category**	**Positive or negative news**	**Topic**	**Number of comments**
2021.1.4-2020.1.6	Safe	Negative	#Shijiazhuang entered a state of war#	3,725
	Material	Negative	#Shijiazhuang residents rush to buy rice, flour, grain, and oil#	431
2021.1.7-2020.1.10	Safe	Negative	#259 positive cases were detected in Gaocheng District of Shijiazhuang#	2,843
			#Shijiazhuang residents stay at home for 7 days#	2,476
	Material	Positive	#70 supermarkets in Shijiazhuang promise not to increase the price of storable vegetables#	1,139
			#Shijiazhuang is offering a maximum reward of 5,000 Yuan for reporting price gouging#	1,385
			#Buying food in Shijiazhuang#	1,537
			#All stores in Shijiazhuang have suspended offline business#	866
			#Shijiazhuang food deliverymen speed up to work#	17
			#Shijiazhuang food deliverymen start work one after another#	87
			#Vegetable Supply in Shijiazhuang#	86

##### Data Preprocessing

With regard to the selected Weibo topics and comments, data preprocessing is conducted first. The first step is to clean up the emojis in the comments. We keep the emojis that can be converted into text like “[heart]” and “[tears],” and delete the emojis that cannot be converted into text. The second step is to eliminate invalid and bot comments. Invalid comments that only contained numbers, punctuation marks, or empty words are deleted. Referring to the representative features of robot accounts pointed out by Loyola et al. (39) suspected bot comments are removed. Finally, 14,449 comment data is selected.

##### Emotion Analysis of Comments

Emotion analysis is carried out on comment data. Emotion dictionary to perform emotion analysis based on Python is used. The dictionary is divided into three parts: emotion dictionary, degree word dictionary, and emoji dictionary.

The specific emotion analysis steps are as follows:

① Perform text preprocessing on a single Weibo sentence, and use punctuation as a segmentation mark to divide a single Weibo into *n* sentences, and extract the emotional word in each sentence.
② Use clauses as the processing unit, look for positive or negative emotional words in the emotional vocabulary, and use each emotional word as a benchmark to look for the degree words in turn, and calculate the corresponding score. Sum up the scores of each emotional word in the clause.
③ Determine whether there are emoticons in the sentence. If so, the clause adds or subtracts the corresponding weight on the basis of the original score.
④ Accumulate the scores of all the clauses of this Weibo to get the final score of this Weibo.

Finally, the emoticon score of each comment is obtained, which is statistically sorted and summarized as shown in Table 3.

**Table 3:** Overall statistic of emotion analysis.

**Statistical items**	**The first stage**		**The second stage**	
	**Safe topic**	**Material topic**	**Safe topic**	**Material topic**
Number of positive comments	1,135	102	2,002	1,752
Number of negative comments	1,429	199	1,893	1,709
Number of neutral comments	1,118	110	1,364	1,636
Proportion of negative emotions	0.39	0.48	0.36	0.34
Ratio of positive and negative comments	0.8	0.5	1.1	1
Average score of positive emotion	1.9	1.8	2.2	1.9
Average score of negative emotion	−2	−2.2	−2.2	−1.9
Total emotional average score	−0.2	−0.6	0.1	0
Average score of positive/negative emotion	1	0.8	1	1
Positive score variance	2.7	2.2	3.6	2.6
Negative score variance	2.6	2.7	2.9	2.3
Total emotion score variance	4.6	4.6	6	4.2
Positive/negative score variance	1.1	0.8	1.3	1.2

People's panic is measured by their negative emotions. It can be seen from the line of “proportion of negative emotions.” From the first stage to the second stage, the proportion of negative comments on Safety News decreased from 0.39 to 0.36, and the proportion of negative comments on material news decreased from 0.48 to 0.34, representing a decline in people's panic. It can be seen from the line of “Average score of negative emotion” that, although the public's concern about safety has increased in the second stage, the average score of negative emotion of safety news has decreased from −2 to −2.2, but the negative emotion of material news has been alleviated, and the average score of negative emotion of material news has increased from −2.2 to −1.9, which also shows the decline of public panic.

##### Measure the Strength of Epidemic Information

In order to simulate the impact of changes in external epidemic information on public panic and panic buying behavior, it is necessary to quantify the intensity of the epidemic information in the case. Topic reading times are the times that netizens read the topic, which can represent the spreading range of the topic. Generally speaking, the higher number of topic readings means the more netizens they see, the more netizens pay attention to this issue. Therefore, the number of topic readings can be used as a measure of topic information intensity. In addition, the number of total comments crawled represents the degree of discussion of the topic by netizens, and can also be used as a measure of the strength of topic information. These two measurement indicators are used to comprehensively quantify the intensity of epidemic information, and the quantitative results are shown in Table 4.

**Table 4:** Information intensity.

**Time**	**2021.1.4-2020.1.6**		**2021.1.7-2020.1.10**	
**News category Positive or negative news**	**Safe Negative**	**Material Negative**	**Safe Negative**	**Material Positive**
Reading times	330 million	5.063 million	340 million	330 million
Information intensity 1	1	0.015	1	1
Number of total comments crawled	3,725	431	5,319	5,117
Information intensity 2	0.745	0.086	1	1
Average information intensity	0.873	0.051	1	1

After statistical analysis, it is found that the highest reading times for material and safety topics in different periods is about 330 million. According to the latest financial report released by Sina Weibo, as of September 2020, the monthly active users of Weibo were 511 million, and the average daily active users were 224 million. Therefore, the range of users affected by 330 million topic readings has been very high. As such, the analysis sets the news information intensity of 330 million or more topics to be read as 1. In turn, the information intensity of other topics is calculated based on 330 million. The calculated information intensity is shown in Table 4 “Information intensity 1” Line. The maximum number of comments on material and safety topics in different periods is about 5,000. Therefore, the information intensity of the epidemic information for the total number of crawled comments of 5,000 and above is set as 1, and the information intensity of other topics is calculated based on 5,000. The calculated information intensity is shown in Table 4 “Information intensity 2” Line.

Finally, the average value of information intensity under the two indicators (i.e., information intensity 1 and 2) is taken as the final value of information intensity of different categories in different periods by combining reading times and number of total comments crawled. The calculated results are shown in the line “Average Information Intensity” in Table 4.

#### Case 2: Panic Buying in UK

In March 2020, affected by the spread of COVID-19, the British experienced a trend of hoarding goods. Many supermarkets in London witnessed panic buying. Toilet paper, hand sanitizer, and canned food were all swept away (40). In December 2020, due to the emergence of new coronavirus variants, the UK announced the implementation of the highest level of “level 4” blockade restrictions on London and the southeast of the UK. As it turned out, some people were worried about the shortage of goods and hoarded goods in supermarkets, which evolved into a panic buying frenzy (41).

In order to study the differences of two panic buying events in the UK in March and December 2020, we took #UK panic buying# and #London panic buying# as keywords to obtain relevant tweets and the comments under the tweets from March 2020 to April 2020 and from December 2020 to January 2021. Finally, we crawled 247 posts and 15,656 comments.

Follow the method in Case 1 for data analysis:

(1) Clear the posts irrelevant to panic buying, and further divide all data into two categories, positive material information and negative material information, and then preprocess the data. Finally, we obtain a total of 157 posts and 8,543 comments.

(2) Through the calculation of sentiment analysis, the proportion of negative emotions rises from 0.178 in the first stage to 0.183 in the second stage, which means that the people's panic has increased.

(3) As for information intensity, because of the long duration of COVID-19, people are gradually numb to the changes in the number of cumulative confirmed cases. Therefore, we use more serious daily deaths to measure the intensity of safety information. According to COVID-19 data released by the WHO website (42), in March 25, 2020, the number of daily deaths increased by 148 in Britain, and in December 22, 2020, the number of daily deaths increased by 215. The highest daily death in the UK in 2020 was 1,105. Taking 1,105 as the benchmark, the negative information intensity of safety in the first stage is 0.13, and in the second stage is 0.19. Next, we measured material information intensity by the number of comments crawled. The highest total number of comments is about 3,500. Therefore, the information intensity of materials with a comment number of 3,500 or above is set as 1, and other information intensity is calculated on the basis of 3,500. After calculation, in the first stage, the positive information intensity of material is 0.34 and the negative information intensity of material is 0.67. In the second stage, the positive information intensity of material is 0.36 and the negative information intensity of material is 1.

### Case Simulation

**Figure 11:**
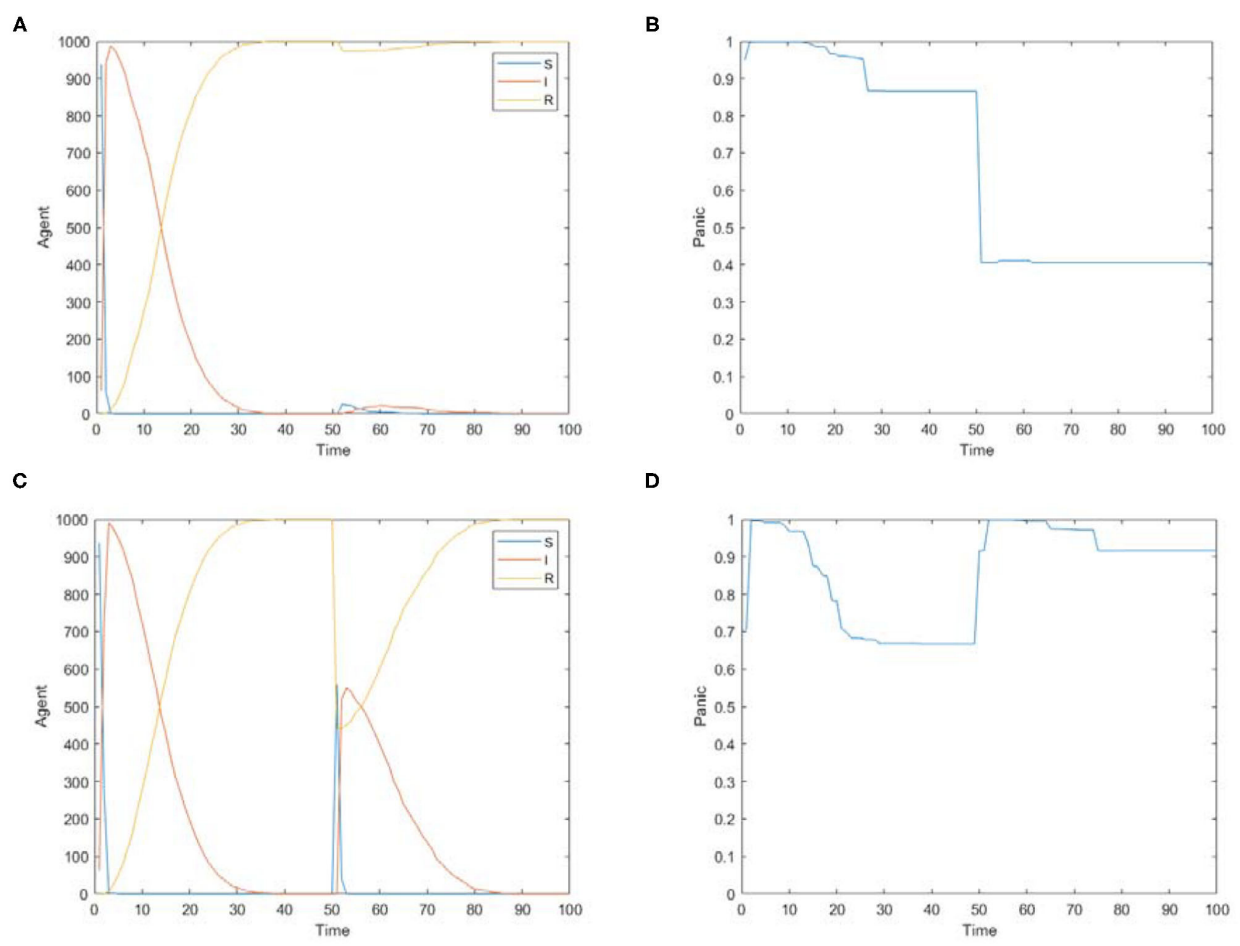
The simulations of two cases using the model proposed in this paper. **(A)** The distribution of panic buyers over time in case 1 simulation. **(B)** The change of average panic emotion over time in case 1 simulation. **(C)** The distribution of panic buyers over time in case 2 simulation. **(D)** The change of average panic emotion over time in case 2 simulation.

Due to the large amount of data, considering comprehensive visualization, the simulated network scale is set to 1,000. Since panic buying behavior was originally caused by external information, based on the above analysis of information intensity, the information intensity in the experiment is set as follows: For the first case (in China), in the first stage, the information intensity of negative and positive safety news is set as *I*_*S*−_(*t*) = 0.873, *I*_*S*+_(*t*) = 0, and the intensity of negative and positive material news is *I*_*M*−_(*t*) = 0.051, *I*_*M*+_(*t*) = 0. In the second stage, the intensity of negative and positive safety news is *I*_*S*−_(*t*) = 1, *I*_*S*+_(*t*) = 0, and the intensity of negative and positive material news is *I*_*M*−_(*t*) = 0, *I*_*M*+_(*t*) = 1. For the second case (in the UK), in the first stage, *I*_*S*−_(*t*) = 0.13, *I*_*S*+_(*t*) = 0, *I*_*M*−_(*t*) = 0.67, *I*_*M*+_(*t*) = 0.34. In the second stage, *I*_*S*−_(*t*) = 0.19, *I*_*S*+_(*t*) = 0, *I*_*M*−_(*t*) = 1, *I*_*M*+_(*t*) = 0.36. In both cases, the other parameter settings are the same: the individual conformity degree *Con*(*i*) obeys the normal distribution of N~(0.5, 0.15), and is mapped to [0, 1], indicating that the conformity degree of most individuals is general; parameter *a* of the material needs (physiological needs) is 0.6 and parameter *b* of safety need is 0.4. Setting the proportion of individuals (i.e., infected people *I*) is 6%, and the remaining individuals are all susceptible *S*; μ_1_ = 0.2, μ_2_ = 0.2, *d*_1_ = 0.2, *d*_2_ = 0.6, *c*_1_ = 1, *c*_2_ = 0.01. The simulation results are shown in Figure 11.

Figure 11 is the simulation figures of two cases using the model proposed in this paper, in which Figures 11A,C simulates the distribution of panic buyers over time in case 1 and case 2, respectively, and Figures 11B,D simulates the change of average panic emotion over time in in case 1 and case 2, respectively.

It can be seen from Figure 11A that there have been two-stage changes in panic buyers in case 1. In the first stage when Time∈[0, 50], the number of panic buyers rises rapidly with most individuals participating in the panic buying, and then the number of panic buyers declines. In the second stage when Time∈[50, 100], the number of panic buyers increases slightly with only a few individuals participating in the material panic buying, and then the number of panic buyers declines. The change curve of infected people *I* (people who participated in the panic buying) in Figure 11A is basically the same as the data change trend of the real case in Figure 10.

It can be seen from Figure 11B, in case 1, when Time∈[0, 50], the average value of panic first rises and then drops to about 0.87 and remains stable. With the continuous reports of positive news related to materials, when Time∈[50, 100], the average value of panic drops. However, due to the severe COVID-19 situation, the average value of panic does not drop to 0, but stabilizes at about 0.4. The declining trend of the panic emotion in the second stage in Figure 11B is the same as the emotion trend of the real case in Table 3.

It can be seen from Figure 11C, in case 2, there are also two-stage changes in panic buyers. In the first stage, the number of panic buyers increased rapidly—almost everyone participated in the panic buying—and then the number of panic buyers decreased. In the second stage, half of the individuals participated in the panic buying, and then the number of panic buyers decreased. According to the relevant retail data of the British Bureau of statistics (43), in March 2020, the Relative Strength Index (RIS) of food stores in March 2020 increased by 9.3% year-on-year, while medical and toilet goods increased by 3.4% year-on-year. In December 2020, the RSI of food stores increased by 4.5% year-on-year, and medical and toilet goods decreased by 0.4% year-on-year. This shows that the quantity of materials purchased in March was higher than that in December, which reflects that the panic buying situation in March was more serious than that in December. Therefore, the real data situation is similar to the simulation results in the figure.

It can be seen from Figure 11D, in case 2, when Time∈[0, 50], the average value of panic first rises and then drops to about 0.67. With the upgrading of blocking measures, when Time [50, 100], the average value of panic rises again, and then drops to about 0.9. The comparison shows that people's panic emotion is higher in the second panic buying, which is similar to the trend of real emotion score in case 2.

It can be found from these two cases that although the environments of the cases are not the same, the simulation results of the cases are relatively close to the real situation, which shows that the panic buying model proposed in this paper can simulate panic buying events in different situations. This model has good applicability and effectiveness and it has important guiding significance for analyzing the causes of panic buying and predicting the changing trend of panic buying.

## Conclusion

This study aims to quantitatively explore the formation and propagation mechanism of panic buying under a sudden epidemic. Therefore, this paper constructs a panic buying propagation model based on SIR model and discuss the influence of individual needs, panic, individual conformity, interaction number, and released time of external information, and verifies feasibility and effectiveness of model by two empirical cases.

The following conclusions are obtained through simulation experiments:

1. The dissipation rate of individual panic is related to the number of people interacting with it, but it is not that the more or less people interacting with, the faster the individual panic will dissipate. There is a threshold. When the number of individuals interacting with each other reaches this threshold, the panic will dissipate the fastest.
2. The released time of the external information will have an impact on the occurrence of a second wave of panic buying. Releasing information of sufficient supplies at the same time as the information of epidemic escalation can help avoid second panic buying. When the first wave of panic buying is coming to an end, it is better to curb the size of the second rush by sending out positive messages after the first panic buying than ahead of the end.
3. Higher conformity among people escalates panic, resulting in panic buying.

However, this paper still has the following shortcomings, which need further study:

1. Although the impact of relevant epidemic information on individual needs is mentioned in the model, it does not identify the publisher of external information sources, that is, it does not consider the difference between information released by government agencies or mass media or netizens. In follow-up research, it can be further refined and improved.
2. The model does not take into account the degree of individual trust in external information sources. In different countries and regions, people's trust in government officials may be different, which will also lead to different perceptions of materials and safety news. In the follow-up research, it is necessary to consider the individual's trust in the information source.

## Data Availability Statement

The raw data supporting the conclusions of this article will be made available by the authors, without undue reservation.

## Author Contributions

PF designed the study and conceived the manuscript. BJ implemented the simulation experiments. TC and CX drafted the manuscript. JY and GC were involved in revising the manuscript. All authors were involved in writing the manuscript and approve of its final version.

## Conflict of Interest

The authors declare that the research was conducted in the absence of any commercial or financial relationships that could be construed as a potential conflict of interest.
